# The pathogenesis of cingulate atrophy in behavioral variant frontotemporal dementia and Alzheimer’s disease

**DOI:** 10.1186/2051-5960-1-30

**Published:** 2013-07-05

**Authors:** Rachel H Tan, Karen Pok, Stephanie Wong, Daniel Brooks, Glenda M Halliday, Jillian J Kril

**Affiliations:** 1 Neuroscience Research Australia, Barker Street, Randwick, Sydney, NSW 2031, Australia; 2 Discipline of Pathology, Sydney Medical School, The University of Sydney, Sydney, NSW 2206, Australia; 3 School of Medical Sciences, University of New South Wales, Sydney, Australia; 4 Discipline of Medicine, Sydney Medical School, The University of Sydney, Sydney, Australia

**Keywords:** Anterior cingulate cortex, Posterior cingulate cortex, Behavioral variant frontotemporal dementia, Alzheimer’s disease, Tau, TDP-43

## Abstract

**Background:**

Early atrophy of the cingulate cortex is a feature of both behavioral variant frontotemporal dementia (bvFTD) and Alzheimer’s disease (AD), with degeneration of the anterior cingulate region increasingly recognized as a strong predictor of bvFTD. The total number of neurons in this region, rather than the density of neurons, is associated with mood disturbance in other dementias, although there are no data on the extent and magnitude of neuronal loss in patients with bvFTD. While the density of small populations of neurons in this region has been assessed, it is unlikely that the degree of atrophy of the cingulate cortex seen in bvFTD can be explained by the loss of these subpopulations. This suggests that there is more generalized degeneration of neurons in this region in bvFTD.

The present study assesses total neuronal number, as well as characteristic pathologies, in the anterior and posterior cingulate cortices of pathologically confirmed bvFTD (N = 11) and AD (N = 9) patients compared with age-matched controls (N = 14). The bvFTD cohort comprised 5 cases with tau pathology (Pick’s disease), and 6 with TDP-43 pathology.

**Results:**

At postmortem, atrophy was detected in the anterior and posterior cingulate cortices of bvFTD cases, but only in the posterior cingulate cortex of AD cases. As predicted, there was a significant reduction in both the density and total number of neurons in the anterior but not the posterior cingulate cortex of bvFTD cases with the opposite observed for the AD cases. Importantly, neuronal loss in the anterior cingulate cortex was only observed in cases with tau pathology.

**Conclusions:**

This study confirms significant neuronal loss in the posterior but not anterior cingulate cortex in AD, and demonstrates that significant neuron loss in bvFTD occurs only in the anterior cingulate cortex but only in cases with tau pathology compared with cases with TDP pathology. We propose that significant neurodegeneration in the anterior cingulate cortex may be useful in differentiating the pathological subtypes *in vivo*.

## Background

Structural and metabolic neuroimaging studies have shown that the cingulate cortex is one of the earliest affected regions in both the behavioral variant of frontotemporal dementia (bvFTD) and Alzheimer’s disease (AD), with the seemingly reverse pattern of cingulate involvement potentially helpful in the differentiation of these two dementia syndromes [1,2]. Atrophy of the anterior cingulate cortex (AC) is increasingly recognized as a strong predictor of bvFTD [2,3], and the aberrant social behavior and affective changes typical of this dementia have been ascribed to the selective vulnerability of von Economo neurons (VENs) within this region [4]. However, VENs constitute only 1-2% of the total neurons in cortical layer 5b in the AC, deeming it improbable that the selective destruction of these neurons alone can account for the severe and early atrophy of the AC observed in bvFTD. Similarly, a loss of VENs has been reported in AD [5], a dementia in which behavior is relatively preserved until late in the disease. To date, histopathological analyses of the cingulate cortex in bvFTD and AD have quantified the density of specific neuronal populations or laminae [4,6]. There has been no assessment of the entire neuronal population of the cingulate cortex in either disease. Furthermore, as there is significant atrophy of the cingulate cortex in both bvFTD and AD the magnitude of the neuronal loss may have been underestimated. The quantification of total neuron number in cortical regions requires accurate measurement of the volume of the region [7]. The significance of assessing total neuronal loss has been emphasized in Huntington’s disease, where mood symptomatology associates with total neuronal loss, rather than the loss of a specific neuronal population in the AC [8]. The present study assesses total neuron numbers in the AC and posterior cingulate cortex (PC) in pathologically confirmed cases of bvFTD and AD to determine the pathological basis of neuroimaging findings observed in these dementias.

## Results and discussion

### Demographics

The present study was comprised of 11 cases with a clinical and pathological diagnosis of bvFTD (FTLD-TDP: n = 6; FTLD-tau: n = 5), 9 cases with clinical and pathological AD, and 14 age-matched controls. Although bvFTD and AD cohorts did not differ in terms of age or sex distribution (all p values > 0.1) (Table 1), multivariate analysis demonstrated an interaction between neuronal loss and age (p < 0.05). Therefore age was included as a covariate in the analyses.

**Table 1:** Group demographics and pathological variables (standard deviation) for patients with behavioral variant frontotemporal dementia (bvFTD), Alzheimer’s disease (AD) and controls

	**bvFTD**	**AD**	**Control**
**N (% male)**	11 (6)	9 (7)	14 (9)
**Mean age at death (year)**	67 (9)	73 (5)	73 (10)
**Postmortem delay (hour)**	9 (12)	14 (10)	24 (12)
**Mean age of diagnosis (years)**	59 (5)	68 (5)	-
**Duration of disease (years)**	7 (4)	5 (2)	-
**Clinical dementia rating (0 – 3)**	2.2 (0.9)	1.7 (0.9)	0

### Volumes

**Figure 1:**
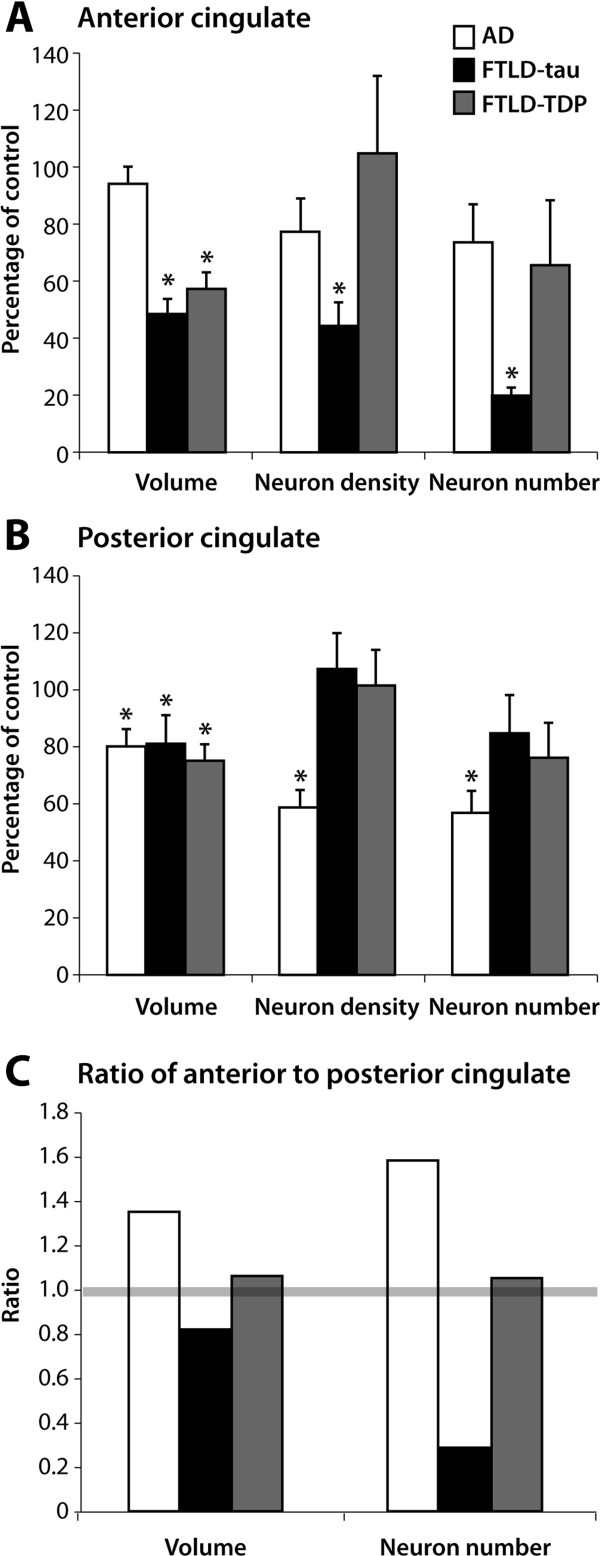
**Degeneration of the anterior and posterior cingulate cortex in AD and bvFTD.** Mean volume, neuron density and total neuron number (± standard error) in Alzheimer’s disease (AD), FTLD-tau and FTLD-TDP expressed as a % of mean control values in the anterior cingulate **(A)** and posterior cingulate **(B)**. The extent of degeneration in the anterior cingulate compared to the posterior cingulate in AD, FTLD-tau and FTLD-TDP is identified as a change from an equal ratio identified by grey bar at 1.0 **(C)**. *p < 0.05 in comparison to controls.

Significant volume loss was observed in AC in cases with bvFTD compared to controls and AD (p < 0.001, Figure 1A). In PC, volume loss was significant in both bvFTD and AD (p < 0.5, Figure 1B). No differences were observed in the degree of atrophy between bvFTD cases with either tau or TDP-43 pathology (p > 0.05, Figure 1). Assessment of the AC:PC volume ratio revealed a reduced ratio for bvFTD cases with tau pathology, no change in the ratio for bvFTD cases with TDP-43 pathology, and an increased ratio in AD cases (Figure 1C).

### Neuronal loss

Significant between-group differences in regional neuronal densities and estimated neuronal numbers were identified (p < 0.001), with changes in neuronal density largely reflecting the degree of degeneration in total neuronal numbers (Figure 1). In the AC, neuronal loss was only significant in bvFTD cases compared with both controls (means ± standard deviation total number of 55,555,584 ± 21,086,167, bvFTD 39 ± 10% of mean control values, p < 0.05) and AD (74 ± 11% of mean control values, p = 0.05), with the loss observed only in cases with FTLD-tau (45% more neuronal loss in AC than those with FTLD-TDP, p < 0.05, Figure 1A). Despite similar PC atrophy in all groups (Figure 1B), only cases with AD had significant neuronal loss compared with controls (mean total number of 46,684,395 ± 18,466,349, Figure 1B). There were no significant differences between the PC neuronal number in the bvFTD subgroups (p > 0.05, Figure 1B). Assessment of the AC:PC neuronal number ratio revealed a more significantly reduced ratio for bvFTD cases with tau pathology, no change in the ratio for bvFTD cases with TDP-43 pathology, and a more significantly increased ratio in AD cases compared with the volume ratios in the same regions (Figure 1C).

### Neuronal pathologies

**Figure 2:**
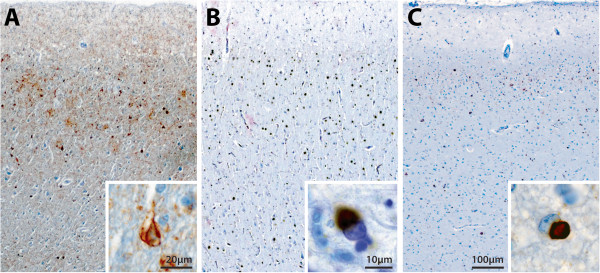
**Photomicrographs of the posterior cingulate cortex (PC) in Alzheimer’s disease (AD, A) and the anterior cingulate cortex (AC) in FTLD-tau (B) and FTLD-TDP (C).** Neuronal loss is obvious in these regions, with more severe degeneration observed in FTLD-tau **(B)**, and a similar extent of degeneration observed in FTLD-TDP **(C)** and AD **(A)**. Comparable levels of tau-immunopositivity in neurofibrillary tangles **(A**, inset**)** and Pick bodies **(B**, inset**)** was observed in PC in AD **(A)** and in AC in FTLD-tau **(B)**. Sparse numbers of TDP-43 inclusions **(C**, inset**)** were found in cases with FTLD-TDP **(C)**.

No pathological deposits were found in the cingulate cortex of any control. TDP-43-immunoreactivity in FTLD-TDP cases was sparse in the cingulate cortex despite typical histopathology in the frontal, entorhinal and temporal cortices of these cases. TDP-43-immunoreactive inclusions consisted of small, dense and rounded neuronal cytoplasmic inclusions (Figure 2C). TDP-43-immunoreactive dystrophic neurites were mostly short and fine, with only one case demonstrating sparse additional long dystrophic neurites. Abnormal tau deposition as intracellular neurofibrillary tangles and Pick bodies was observed in the cingulate cortex in all cases with AD and FTLD-tau, respectively (Figure 2A-B). Tau-immunoreactive dystrophic neurites were also observed in all AD and FTLD-tau cases. In FTLD-tau, the density of tau inclusions was greatest in the AC (mean ± SE of total remaining neurons: 24.5% ± 5.7%) compared to PC (mean ± SE of total remaining neurons: 2.3% ± 0.6%, p = 0.001). This regional difference in pathological severity was not observed in either FTLD-TDP (mean ± SE of total remaining neurons: AC: 19 ± 4.4; PC: 24 ± 22) or AD (mean ± SE of total remaining neurons: AC: 2 ± 0.8; PC: 0.6 ± 1.4). Significant regional differences between groups were observed in FTLD-TDP compared to FTLD-tau (p < 0.005) but not AD (p > 0.05).

### Pathology correlations

Spearman rank correlations were used to determine any relationships between the severity of pathological inclusions and the degree of neuronal loss in the AD and bvFTD sub-groups. These analyses revealed an association between the severity of neuronal loss with pathology in AD (ρ = −0.614, p = 0.007) and FTLD-tau (ρ = −0.842, p = 0.002), but not in FTLD-TDP cases.

### Clinicopathological correlations

Spearman rank correlations were also used to identify any relationships between regional neuronal loss or the percentage of inclusion pathology with disease duration and last Clinical Dementia Rating score in the AD, bvFTD and bvFTD subgroups. The severity of pathological inclusions increased with an earlier onset of AD (ρ = −0.638, p < 0.005). No other associations were identified.

### Discussion

This is the first quantitative analysis of total neuronal number in the cingulate cortex of bvFTD and AD that accounts for volumetric changes in this region. The degree of cingulate atrophy demonstrated in structural imaging suggests considerable degeneration of neurons in both dementia groups, which our data supports. However, direct comparison between patient groups showed significant AC neuronal loss only in bvFTD and only in cases with Pick disease pathology, while we can confirm significant PC neuronal loss occurs in AD [9]. We can also add that overall neuronal numbers in the PC are largely spared in pathologically confirmed cases of bvFTD, despite atrophy of this region by end-stage. Our data are consistent with previous volumetric data showing atrophy of PC over the course of bvFTD and not at initial diagnosis across all pathological subtypes [3] or in cases with later confirmed FTLD-TDP [10,11]. This information suggests that later PC atrophy in bvFTD most likely reflects synaptic loss and/or cell shrinkage, as we did not observe significant total neuronal loss in the bvFTD cases with PC atrophy.

In bvFTD, neuronal loss in AC was observed only in cases with FTLD-tau (Pick bodies) compared to those with FTLD-TDP. This differs from other regional changes where the hippocampus is more affected in patients with FTLD-TDP compared with FTLD-tau [3], with the suggestion that more severe episodic memory and hippocampal atrophy may be a potential biomarker for FTLD-TDP *in viv*o. The present data now show variability in the populations of neurons vulnerable to tau pathology in bvFTD and may suggest that greater neuronal loss and atrophy in AC could reflect underlying FTLD-tau, the presence of Pick bodies specifically, in *in vivo* studies. Of significance is our previous finding that AC is affected early in bvFTD while atrophy of PC is a late event [3]. This suggests that rapid AC atrophy could be tested as an early biomarker for FTLD-tau, although this finding would need to be confirmed in other tauopathies as only Pick disease was included in the present study.

## Conclusions

BvFTD is a clinically heterogeneous disorder and correlations between clinical and pathological subtypes have been weak at best [12]. The need to distinguish between the underlying pathologies is becoming increasingly apparent with increasing efforts directed towards the development of targeted disease-modifying therapies in FTD. For many years drugs targeting tau protein abnormalities have been investigated as a potential treatment for AD. However, given the occurrence of both tau and Abeta pathologies in AD, the therapeutic effects of tau-directed treatments may be less obvious in AD than when assessed in patients with pure pathology such as seen in FTLD-tau (see [13] for a review). While future studies comparing the extent of neurodegeneration across larger pathological subgroups are necessary, the present findings suggest that greater atrophy of AC may predict FTLD-tau, a finding worthy of further clinicopathological validation.

## Methods

### Case selection

Eleven cases with a clinical diagnosis of bvFTD [14] and FTLD pathology [15], 9 cases with clinical and pathological AD [16], and 14 controls without dementia or significant neuropathological abnormalities were selected from a neuropathological series of cases collected by the Sydney Brain Bank through a regional brain donor program. Patients were diagnosed by experienced clinicians using standard diagnostic criteria [17,18] following a medical interview and an informant history. The bvFTD all had changes in personality or social behaviour (eg. apathy, disinhibition, stereotypic behaviours, alterations in food preference, or poor self-care) and executive dysfunction (eg. poor planning, forethought, reasoning or organisation). The program holds approval from the Human Research Ethics Committee of The University of New South Wales and complies with the statement on human experimentation issued by the National Health and Medical Research Council of Australia. This research project was approved by the Human Research Ethics Committees of the Universities of Sydney and New South Wales. Some of the cases included in this study have been reported in previous publications [3,19,20]. The research program used standardized tests following the patients longitudinally with the last assessments performed within 14 months of death. All dementia cases had Clinical Dementia Rating (CDR) scores between 1 and 3 while controls had scores of <0.5. The postmortem interval was 16 hours on average (range: 2–45 h; mean ± SD for bvFTD = 9 ± 12, for AD = 14 ± 10, and for control = 24 ± 12, ANOVA p > 0.05). Cases with bvFTD with TDP pathology (FTLD-TDP, n = 6, 5 males; age range 53–72 years; 2 with type A, 2 with type B and 2 with type C) [21] and tau pathology (FTLD-tau, n = 5, 1 male; age range 65–79 years; 5 with Pick’s disease) were selected for this study. Cases with all types of TDP-43 pathologies were selected, as our previous study showed no differences between different TDP-43 pathology types at end-stage [3]. Case details are shown in Table 1.

### Tissue preparation and volume measurements

Tissue preparation and volumetric methods used in this study have been published in detail elsewhere [19,20]. Briefly, brains were collected at autopsy, fixed in 15% neutral buffered formalin for two weeks, the fixed brain weighed and the volume determined by fluid displacement. The cerebellum and brainstem were separated from the cerebrum by sectioning through the cerebral peduncles. Each cerebrum was embedded in 3% agarose, sectioned in 3 mm coronal slices using a rotary slicer, photographed and printed at 1× magnification. The average slice thickness for each brain was determined by dividing the hemisphere length by the total number of slices. Diagnostic neuropathological screening was conducted using standardized blocks taken from the frontal, parietal, temporal and occipital cortices, amygdala, hippocampus, basal ganglia, diencephalon, midbrain, pons, medulla oblongata and cerebellum. Examination of sections stained with haematoxylin and eosin and modified Bielschowsky silver stain, as well as immunohistochemically for tau, ubiquitin, α-synuclein and TDP-43, confirmed pathological diagnoses.

Anatomical structures and gyral boundaries most consistently associated with cytoarchitectonic boundaries were used to identify the AC and PC for all cases, as previously described [19,20]. The AC was divided from the PC at the central lobule of the central sulcus. The volumes of these regions were determined by a point counting procedure on the brain slice photographs as previously described [19,20]. Briefly, the areas corresponding to each region were identified on the brain slice photographs and were randomly overlaid with a grid of 3848 points (each separated by 4 mm). The total number of points falling on each region of interest was counted and the volumes calculated by multiplying the sum of points falling on a given region by the volume represented by each point (volume/point = 16 mm^2^ × mean slice thickness; average of 50 mm^3^). This method is routinely used in our laboratory to measure regional volumes postmortem [20,22,23] and approximates current point counting procedures used in MRI studies of brain volumes.

### Quantitation of neuron numbers and inclusion pathologies

Standardised tissue samples of the cingulate cortices were sampled (AC just posterior to the genu of the corpus collosum, PC just anterior to the splenium of the corpus callosum) and embedded in paraffin wax using routine procedures. Ten μm serial sections were cut from each block and immunohistochemically stained with antibodies to tau (mouse anti-human tau diluted 1:1000; Thermo Scientific) and TDP-43 (rabbit anti-human TDP-43 diluted 1:800; ProteinTech) using routine techniques [3]. An additional section was stained with cresyl violet (0.5%). Quantitation of cortical neuronal populations was performed as previously described and validated [7]. Briefly, 2 × 500 μm wide strips through the entire cortical thickness from the pial surface to white matter were randomly sampled by marking perpendicular lines on the coverslip of each slide. Strips were not selected if the cortex was cut obliquely at the marked line to ensure even representation of all cortical laminae. Neurons were counted at 200× magnification using a 10 × 10 eyepiece graticule (500 μm × 500 μm) with standard inclusion (lower and left) and exclusion (upper and right) borders in contiguous, non-overlapping fields. The density of neurons within each region was calculated for each case, and the total number of neurons estimated by multiplying neuronal density by the calculated volume for the AC and PC, as appropriate. The percentage of neurons with cytoplasmic inclusions in the AC and PC was expressed as a percentage of total remaining neurons. Quantitation was performed by two raters blind to case details with an inter-rater variance of 2.4% and intra-rater variability of 2.2%.

### Statistical analysis

Data were analysed using SPSS19.0 (IBM Corp., Chicago, Ill., USA). Group differences in age and postmortem delay across all groups were investigated using analysis of variance (ANOVA), and where these variables showed significant differences, they were entered as co-variates in the multivariate ANOVA. Post hoc analyses using the Bonferroni correction for multiple comparisons were performed to identify specific group differences in regional neuronal loss. Any associations between neuronal number and the percentage of neurons with pathological depositions (tau, TDP-43) or measures of disease severity (disease duration and CDR score) were identified with Spearman rank correlations.

## Abbreviations

AC: Anterior cingulate; PC: Posterior cingulate; AD: Alzheimer’s disease; bvFTD: Behavioral-variant frontotemporal dementia; VENs: Von Economo neurons.

## Competing interests

The authors declare that they have no competing interests.

## Authors’ contributions

RT, GH and JK were responsible for the design of the study. GH and JK characterized the cases for inclusion in the study. KP, SW, DB and RT undertook the acquisition of data. RT and GH performed the statistical analyses and drafted the manuscript. All authors read and approved the final manuscript.
